# Traditional Chinese Medicine as a Potential Source for HSV-1 Therapy by Acting on Virus or the Susceptibility of Host

**DOI:** 10.3390/ijms19103266

**Published:** 2018-10-20

**Authors:** Wen Li, Xiao-Hua Wang, Zhuo Luo, Li-Fang Liu, Chang Yan, Chang-Yu Yan, Guo-Dong Chen, Hao Gao, Wen-Jun Duan, Hiroshi Kurihara, Yi-Fang Li, Rong-Rong He

**Affiliations:** 1 Guangdong Engineering Research Center of Chinese Medicine & Disease Susceptibility, Jinan University, Guangzhou 510632, China; liwen410@stu2017.jnu.edu.cn (W.L.); wxh1994@stu2016.jnu.edu.cn (X.-H.W.); zorro@stu2015.jnu.edu.cn (Z.L.); lifangl1993@stu2016.jnu.edu.cn (L.-F.L.); yanchang@stu2014.jnu.edu.cn (C.Y.); hichangyu@stu2017.jnu.edu.cn (C.-Y.Y.); chgdtong@jun.edu.cn (G.-D.C.); tghao@jnu.edu.cn (H.G.); duanwj@jnu.edu.cn (W.-J.D.); Hiroshi_Kurihara@jnu.edu.cn (H.K.); 2 Institute of Traditional Chinese Medicine and Natural Products, College of Pharmacy, Jinan University, Guangzhou 510632, China

**Keywords:** herpes simplex virus type 1, traditional herbal medicine, traditional Chinese medicine, natural products, extracts, acyclovir, susceptibility

## Abstract

Herpes simplex virus type 1 (HSV-1) is the most common virus, with an estimated infection rate of 60–95% among the adult population. Once infected, HSV-1 can remain latent in the host for a lifetime and be reactivated in patients with a compromised immune system. Reactivation of latent HSV-1 can also be achieved by other stimuli. Though acyclovir (ACV) is a classic drug for HSV-1 infection, ACV-resistant strains have been found in immune-compromised patients and drug toxicity has also been commonly reported. Therefore, there is an urge to search for new anti-HSV-1 agents. Natural products with potential anti-HSV-1 activity have the advantages of minimal side effects, reduced toxicity, and they exert their effect by various mechanisms. This paper will not only provide a reference for the safe dose of these agents if they are to be used in humans, referring to the interrelated data obtained from in vitro experiments, but also introduce the main pharmacodynamic mechanisms of traditional Chinese medicine (TCM) against HSV-1. Taken together, TCM functions as a potential source for HSV-1 therapy by direct (blocking viral attachment/absorption/penetration/replication) or indirect (reducing the susceptibility to HSV-1 or regulating autophagy) antiviral activities. The potential of these active components in the development of anti-HSV-1 drugs will also be described.

## 1. Introduction

### 1.1. Herpes Simplex Virus Type I (HSV-1) and Related Disease

HSV-1 is a linear double-stranded DNA virus, surrounded by a capsule around the virus core-shell [1]. HSV-1 is prevalent in human being and human is their sole natural host. At present, approximately 100 species have been identified. According to epidemiological surveys, the global prevalence for HSV-1 in people aged 0–49 years was 3.7 billion (roughly 67%) in 2012 [2]. HSV-1 mainly infects skin, lip mucosa, eyes, nervous system, and occasionally the external genitals, thus it can cause a variety of diseases, such as herpes labialis, herpes keratitis, and encephalitis neonatorum, etc. In addition, HSV-1 can establish latent infections, which can be reactivated to cause clinical symptoms under certain conditions, such as psychological and physiological stress [3,4,5,6,7], fatigue [8], ultraviolet irradiation [9,10], physical trauma [11], abnormal hormone levels [12,13,14,15], and immunosuppression [16]. Thus, HSV-1 is so hard to be remedied. Moreover, HSV-1 is more than a common viral infection. Reports have uncovered that HSV-1 acts as a risk factor for Alzheimer’s disease (AD) [17,18,19]. Accumulation of amyloid-beta (Aβ) and AD-like tau (P-tau) were observed in HSV-1 infected cells. Related mechanisms revealed that HSV-1 infection caused the production of several APP fragments (including APP intracellular domain, AICD). AICD was a key factor in the induction of AD by HSV-1 infection, as AICD could bind to the promoter regions of both neprilysin (NEP, the major Aβ-degrading enzyme) and GSK3β (the enzyme responsible for the hyperphosphorylation of tau). Moreover, the activation of pGSK3 caused by HSV-1 infection was essential for the phosphorylation of APP at Thr 668, leading to the intraneuronal accumulation of Aβ. Additionally, HSV-1 infection reduced the expression of synapsin-1 and synaptophysin, and depressed synaptic transmission [20,21]. Consequently, it is meaningful to search for different therapeutic strategies and drugs to suppress HSV-1, especially to prevent the reactivation of latent HSV-1.

TCM (traditional Chinese medicine) theory demonstrated that pathogenic “fire” derived from stagnation of liver-Qi is a stress-induced physiological response involving the central nervous system, endocrine system, and immune system [22]. These changes increase one’s susceptibility to disease. Persistent emotional stress is an important factor for physical and mental fatigue as well as a weakened immunity system and ultimately high susceptibility to HSV-1 [23]. It has been shown that HSVs are the causative agents for cold sores, fever blisters of the mouth (HSV-1) [24], and genital herpes (HSV-2) [25]. Interestingly, recurrent facial “hot sores” mentioned in TCM theory matches the clinical description of HSV-1 infection. Considering many TCM has the function of “clearing liver-fire”, it is therefore of great significance to develop the screening and evaluation of the efficacy of TCM against HSV-1.

### 1.2. Mechanism and Defects of Current Anti-HSV-1 Drugs

There are many ways to treat a viral infection. On the one hand, infection can be treated by interfering with the virus itself, such as direct inhibition or killing of the virus, interference of virus adsorption, prevention of virus penetrating cells, and inhibition of virus biosynthesis and release. On the other hand, treatment can boost the antiviral capacity of the host, so that the host has the ability to kill the virus via its own immune system. The effect of antiviral drugs is mainly achieved by interfering with one of the stages in the virus replication cycle. The current antiviral drugs can be divided into the following categories based on their mechanisms of action: Penetration and dehulling inhibitors, DNA polymerase inhibitors, reverse transcriptase inhibitors, protein inhibitors, neuraminidase inhibitors, and broad-spectrum antiviral drugs.

The main therapeutic drugs frequently used in clinical practice are nucleoside analogues represented by ACV, which affects the virus mainly by affecting the DNA replication process. Although ACV is very effective to treat HSV-1 infection, it is likely for the virus to develop into drug-resistant strains with long term use, due to mutations of DNA polymerase or mutational thymidine nucleoside kinase [26]. The mutagenicity of some nucleoside analogues are high, such as ganciclovir, and hence less safe to use. Moreover, HSV vaccine research is mainly aimed at genital herpes. Yet the vaccine is not effective against genital herpes, let alone other HSV infections. Therefore, it is of great significance to develop new anti-HSV-1 drugs with lower toxicity by acting via a different mechanism compared to the nucleoside analogue. The issue of drug resistance should also be tackled; the drugs to be developed should not introduce a selective pressure that induces the virus to evolve into drug resistant strains.

## 2. Natural Products as Potential Resources for New Antiviral Drugs

Natural products have considerably more structural and chemical diversities than artificially synthesized small molecules. Although natural products may not eventually become new chemical entities that can be listed, they are still used regularly as structural sources for new compounds. Non-synthetic new compound entities accounted for 62% of small molecular compounds from 1981 to 2002 in the cancer field. These molecules can be used as drugs, and continue to be discovered in the field of chemistry, biology, and medicine. Thus, they are the ideal resources for drug development. Around 60% and 75% of the drugs are derived from natural products in the fields of cancer and infectious disease, respectively [27]. For instance, avermectin and its derivative ivermectin, two natural bacterial derived products, have almost eliminated onchocerciasis and lymphatic filariasis. Artemisinin, another natural product derived from TCM, significantly reduces the mortality rate of malaria patients.

Natural products are also important resources for the development of new antiviral drugs. Active substances against HSV-1 extracted from plants, microorganisms, and animals have attracted widespread interest in the past few decades [28,29,30,31]. The scientific and theoretical values of TCM are increasingly recognized and accepted, since TCM is one of the main sources of natural products. Many valuable drugs have been developed with the aid of TCM and it proves that TCM plays a significant role in drug discovery and development. The fractions/extracts and pure compounds with promising anti-HSV-1 activities isolated from TCM will be discussed in the following section.

## 3. Natural Anti-HSV-1 Products from TCM

TCM originated from China has been playing crucial roles in the prevention and treatment of diseases. Chinese medicine is one of the industries with originality and innovational advantages in China because of a well-established sustainable development, material foundations, and social environment. TCM has made huge progress recently in the research and development of anti-HSV-1 drugs under the guidance of TCM theory.

### 3.1. Extracts with Potential Anti-HSV-1 Activities

Crude extracts obtained from individual parts of plants by various extraction approaches have shown a wide spectrum of antiviral activity, including anti-HSV-1 activity specifically. Extracts classified by different source and chemical class will be discussed. The IC_50_ of the extracts against HSV-1, their corresponding mechanism, and trial level achieved at the date of review submission can be found in Table 1.

Litchi flower ethanolic extract (LFEE) showed satisfactory anti-oxidative and anti-inflammatory activities [43]. In addition, in vitro tests confirmed that lychee flower extract (LFE) could also inhibit proliferation and viral replication of HSV-1 [32]. The aqueous extract of *Moringa oleifera* Lam. Leaves (AqMOL) was also reported to be able to resist HSV-1. This is demonstrated by the experiment in which AqMOL was orally administered at a dose of 300 mg/kg to HSV-1-infected mice three times daily on day 0 to 5 post-infection [44]. *Antrodia camphorate* is widely used in the treatment of liver diseases, cancers, as well as skin infections due to its anti-inflammatory property. Recently, the crude ethanol extract of *Antrodia camphorate* has been found to have the potential to inhibit HSV-1. Whilst fraction A and B being the major fractions in the extract, fraction A exhibited better anti-HSV-1 activity than crude extract, suppressing HSV-1 replication significantly in Vero cells after being infected with HSV-1 for 1 to 4 h [34]. Besides, the dried seeds of *N. nucifera* were collected and their ethanolic extracts were identified as NN-H, NN-E, and NN-B. Nine main subfractions were isolated from the total bioactive fraction, NN-B, by thin-layer chromatography. Among the nine active ingredients, the inhibitory effect of subfraction NN-B-5 on HSV-1 was does-dependent and cell line independent. NN-B-5 reduced the propagation of ACV-resistant HSV-1 (TK^−^ HSV-1 strain), based on the results of a plague reduction assay that the inhibitory efficiency of subfraction NN-B-5 on TK^−^ HSV-1 was 85.9 ± 8.3% at the concentration of 50 μg/mL [36]. 

Many different compounds extracted from the roots of a range of herbs/plants have also exhibited anti-HSV-1 activity. Alkaloids extracted from the roots of *Tripterygium hypoglaucum*, with a yield of about 1%, had better anti-HSV-1 activity in vitro than ACV [36]. Lin et al*.* reported that the crude aqueous and ethanolic extracts (like ursolic acid, apigenin, linalool, etc.) of another well-known medicinal herb, *Ocimum basilicum* (OB), were tested for their antiviral activity. Interestingly, ursolic acid, one of these purified components, exhibited the highest activity against HSV-1, with a selectivity index (SI) of 15.2, using ACV as a positive control (SI = 20.3) [37].

Anti-HSV-1 activity of extracts of almond skins were also evaluated. Almond skin extracts restricted the replication of HSV-1 in a dose-dependent manner. Additionally, NS extracts (natural) were more potent inhibitors of HSV-1 than BS extracts (balanced). Data indicated that almond skin extracts exerted antiviral activity by restricting virus penetration into cells and by inhibiting the virus absorption when the cells were treated with high concentrations of polyphenols extracts from almond skins [38].

Additionally, a frequently used TCM named Yin Chen Hao Tang (YCHT) is widely used to treat acute hepatitis with jaundice. The anti-HSV-1 and anti-HSV-2 activity of the water extract of YCHT were investigated in vitro and the results indicated that YCHT water extract inhibited both viruses in a dose-dependent manner [39].

Methanol extract of *Stephania cepharantha* (root tubers), its CHCl_3_-soluble fraction (alkaloid fraction), and FK-3000 (**31**) (the major alkaloid) were tested for their anti-HSV-1 activity, using a cutaneous HSV-1 infection model in BALB/c mice. Data indicated that alkaloids, including FK-3000 (**31**) from the extract, were responsible for the anti-HSV-1 activity. Unfortunately, FK-3000 (**31**) is cytotoxic and has a narrow therapeutic index [40]. Hence, the development of modified compounds with reduced cytotoxicity and side effects can be a direction for further study.

Briefly, large numbers of natural products, including crude extracts and fractions isolated from plants/herbals have been assessed on their antiviral effects on HSV. All the most promising extracts should be purified. Although these compounds exhibited anti-HSV-1 activities, further evaluation is required to figure out the safe dosage of these agents if they are to be used in humans, by referring to the interrelated data obtained from in vitro experiments. Considering that most of the relevant studies only mentioned the potential anti-HSV-1 activities of those extracts in vitro, future studies are also required to precisely investigate the mechanism of the antiviral activity of these natural compounds and the way to make the most of these compounds. In addition, are there any other compounds with potential anti-HSV-1 activity that have not been identified or isolated?

### 3.2. Pure Compounds Isolated from TCM with Anti-HSV-1 Activities 

Since unauthenticated crude extracts of plants were observed to exhibit anti-HSV-1 activity, several research teams have tried to identify specific compounds that were responsible for this activity. Several research journals have currently reported investigation of the anti-HSV-1 activity of TCM extracts or plant/herbal-derived molecules as seen in Table 2 and Table 3.

A number of published reports over the past few years hypothesized the extracts of *Houttuynia cordata* to be one of the therapeutic possibilities of HSV-1 infection. *H. cordata*, also named “Yu-xing-cao”, is a vegetable crop and has been used widely as an effective TCM for hundreds of years due to its anti-inflammatory [64,65] and antiviral [42,46,50] activities.

Research on the antiviral effect of *H. cordata* were mainly focused on its extracts for a long time. It was reported that hot water extracts of *H. cordata* showed activity against HSV [41]. However, the specific ingredients in the extract were not given in the report. Hence, *H. cordata* was worthy of further investigation. Lee et al. elucidated that *H. cordata* water extracts (HCWEs) inhibited the infection of HSV-1 via blocking viral binding and penetration as well as viral replication. As ingredients in the HCWEs, quercetin (**1**) and isoquercitrin (**2**) inhibited NF-κB activation. In addition, quercetin (**1**) could also inhibit viral entry [42]. 

Purification and separation of *H. cordata* extracts greatly promoted the progress of antiviral research. Norcepharadione B (**3**) isolated from the MeOH extract of *H. cordata* showed good inhibitory activity against the replication of HSV-1 by 46.38 ± 1.06% at the concentration of 100 μM [45]. Anti-HSV-1 activity of flavonoids and alkaloids, main non-volatile oil components of *H. cordata*, have also been reported. All houttuynoids showed a structure comprised of a flavonoid core and one (or two) houttuynin chain(s). In the report by Yao et al., it was discovered that houttuynoids A-E (**4**–**8**), a new class of flavonoids, displayed novel skeletons with unprecedented carbon skeletons and possessed potential anti-HSV-1 activities. Houttuynoids A-E (**4**–**8**) were isolated as a brown amorphous power, with the respective selective index (SI) of anti-HSV-1 activity of **4**–**8** found to be 7.08, 3.15, 10.47, 3.02, and 3.21 [46]. Seventeen flavonoids were subsequently isolated from the extract of the same plant by Yao’s research team, including four newly identified houttuynoids (**10**,**11**,**12**,**13**). Except for having the same structure reported previously, these four houttuynoids had two different characteristics: (i) The flavonoid cores contain rutin, quercetin-3-*O-*α-rhamnosyl-(1→6)-β-galactoside, or other components instead of purely hyperoside; and (ii) the compound was comprised of two inseparable epimers, due to the chiral carbon of hemiketal at *C*-3’. The anti-HSV-1 activity and cytotoxicity of the new compounds were then evaluated, using ACV as a positive control. Houttuynoid G (**10**) and houttuynoid H (**11**) were shown to exhibit better inhibitory activities against HSV-1 than houttuynoid I (**12**) and houttuynoid J (**13**) [48]. Further research on HSV-1 resistance to *H. cordata* found that houttuynoid A (**4**) exhibited strong anti-HSV-1 activity both in vitro and in vivo. This compound **4** inactivated HSV-1 by blocking the fusion of the viral envelope with the plasma membrane, thus inhibiting HSV-1 multiplication and preventing lesion formation in an HSV-1 infected mouse model [50]. Ongoing studies have continued to isolate and identify new *H. cordata* compounds. HPLC-DAD-MS chromatograms of *H. cordata* extract revealed that there might be some new houttuynoids, with two houttuynin units in *H. cordata.* Gao et al. isolated houttuynoid M (**16**), the first example of a houttuynoid with a bis-houttuynin chain tethered to a flavonoid core. The pharmacodynamics analysis of houttuynoid M (**16**) showed that this compound had a good inhibitory effect on HSV-1. Nevertheless, the specific mechanism of the anti-HSV-1 effect was not mentioned [49]. So far, Yao’s research team has isolated, purified, and identified thirteen houttuynoids from the extracts of *H. cordata*. In addition to the houttuynoids reviewed above, houttuynoid F (**9**), houttuynoid K (**14**), and houttuynoid L (**15**) also exhibited anti-HSV-1 activity. The inhibitory effect of houttuynoid F (**9**) at 31.25 μM and houttuynoid K (**14**) and L (**15**) at 50 μM was equivalent to ACV at 200 μM [47].

**Figure 1:**
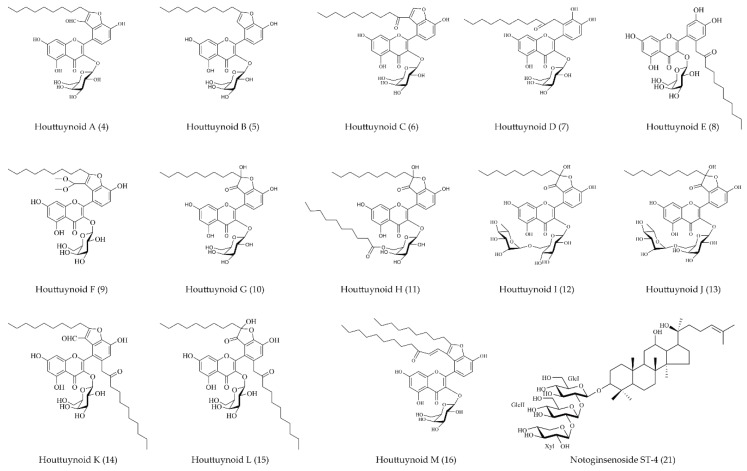
Molecular structure of related compounds. Bold numbers in parentheses refer to the numbers of corresponding compounds.

Though tremendous progress has been made in natural products with anti-HSV-1 activities extracted from *H. cordata*, further questions still need to be addressed. For instance, in the flavonoids of *H. cordata*, the compounds that a lot of efforts have been dedicated to, the active chemical constituents are yet to be identified. It is important to identify the compound that is the main content of the *H. cordata* extract and investigate whether this compound exhibits anti-HSV-1 activity. Additionally, the *H. cordata* plant can be divided into a ground portion and an underground portion. Further research should be conducted to determine the concentration of flavonoids in these portions. Ongoing study will provide a novel insight into the answers to these questions. Though accumulating studies revealed that *H. cordata* extracts could exert anti-HSV-1 activities from multiple machineries, including inhibition of viral binding/penetration/replication and regulation of immune response [42,45,66], whether *H. cordata* extracts exert antiviral effects by regulating autophagy remains to be further demonstrated. The molecular structure of the corresponding compounds can be seen in Figure 1.

The soluble part of *Radix isatidis* (Banlangen), another well-known TCM, was also revealed to be anti-viral. Isoquinoline derivative (**19**), isolated as a component of this extract, showed better anti-viral activity than other constituents. As a result, some analogues have been synthesized chemically and assessed for their inhibitory effects on HSV-1 [51]. Correspondingly, the root of *Strobilanthes cusia* BREMEK (Acanthaceae), popularly known as Da-Ching-Yeh, has been reported to treat influenza, epidemic cerebrospinal meningitis, encephalitis B, viral pneumonia, mumps, and severe acute respiratory syndrome (SARS). Recently, a new feature has been reported because some of its compounds with anti-HSV-1 effects were discovered, like lupeol (**20**), extracted from the root. Although the anti-HSV-1 activity of lupeol (**20**) and its corresponding SI index were lower than those of ACV, lupeol (**20**) was the main component in the extract of *Strobilanthes cusia* root with a proportion of 0.048% [52]. Therefore, lupeol (**20**) was expected to be developed as a natural drug against HSV-1 in the future. A new dammarane-type saponin, named notoginsenoside ST-4 (**21**), was isolated from a famous TCM, the roots of *Panax notoginseng* (Burk.) F.H. Chen (Araliaceae), which was frequently used to remove blood stasis and promote blood circulation (see Figure 1 for the specific molecular structure of ST-4). This is an example of another natural extract that restrains HSV-1 through penetration inhibition [53]. A trend can be seen that more and more extracts from the roots of multifarious TCM have been proved to be effective on HSV-1 infection. Emodin (**22**), extracted from the roots of *Rheum tanguticum,* had anti-HSV-1 activity in vitro and in vivo [54,67,68,69,70]. Furthermore, 1,2,4,6-tetra-*O*-galloyl-β-d-glucose (1246TGG) (**23**), a polyphenolic compound isolated from *Phyllanthus emblica* L. (Euphorbiaceae), was identified to inhibit HSV-1 in vitro [55]. 

*Origanum vulgare* (Lamiaceae), a perennial herb distributed worldwide (Asia, Europe, America, and North Africa), is regularly used in the treatment of the common cold, cough, and indigestion [71]. This plant is also well known for its strong antimicrobial and antioxidant effects due to its phenolic components [72,73]. Reported research has demonstrated that some phenolic compounds showed antiviral potential. Consequently, the phenolic compounds extracted from *O. vulgare* could also be used to evaluate its antiviral evaluation. Indeed, Acacetin-7-*O*-[6′′′-*O*-acetyl-β-d-galactopyranosyl-(1→2)]-β-d-glucopyranoside) (**24**) and 2,5-dihydroxybenzoic acid (**25**) extracted from *O. vulgare* showed weak anti-HSV-1 activity [56].

At the same time, some of the Chinese herbal extracts that are previously reported to be antiviral have also been used to assess their anti-HSV-1 activity. *Plantago major* L., a popular TCM, has been used for virus hepatitis. Lin and coworkers investigated the antiviral activity of water extract and pure compounds of *P. major*, and data indicated that the water extract of *P.* major exhibited minor anti-HSV-1 activity. The pure compound called caffeic acid (**27**), however, showed stronger activity against HSV-1 in a dose-dependent manner. The strongest inhibitory effect of caffeic acid (**27**) against HSV-1 was found within 12 h of infection as long as the concentration was no less than 20 μg/mL [57].

The latest research on isolated compounds reported that the compounds extracted from *Ranunculus sieboldii* and *Ranunculus sceleratus* exhibited anti-HSV-1 activity. *Ranunculus sieboldii* and *Ranunculus sceleratus* are widely distributed in China. They were traditionally used in the treatment of hepatitis B. Nineteen compounds extracted from *Ranunculus sieboldii* and *Ranunculus sceleratus* were tested for anti-HSV-1 activity based on CPE on Vero cells and results showed that protocatechuyl aldehyde (**30**) was the only compound that resisted HSV-1 [58].

The reactivation of HSV-1 in the brain is a potent risk factor of Alzheimer’s disease. It has been reported that curcumin (**35**) had the effect of preventing and treating Alzheimer’s disease [74]. Therefore, the effect of curcumin (**35**) on HSV-1 has been gradually explored. Curcumin (**35**) and its derivatives, like gallium-curcumin (**36**) and Cu-curcumin (**37**), have remarkable anti-HSV-1 effects, with SI values of 14.6, 18.4, and 14.1, respectively [62]. Studies in vitro revealed that curcumin (**35**) exerted antiviral activity by blocking the expression of ICP4 (infected cell polypeptide 4) and ICP27 (infected cell polypeptide 27) (IE genes) [75].

Although the mentioned compounds all reflected their anti-HSV-1 activities, the metabolic pathways of most compounds and the effects of these metabolites on cellular processes were not clear. Therefore, understanding the fate of TCM with anti-HSV-1 activity in vivo will provide strategies for the development of new anti-HSV-1 drugs.

## 4. The Pharmacodynamic Mechanism of TCM against HSV-1

**Figure 2:**
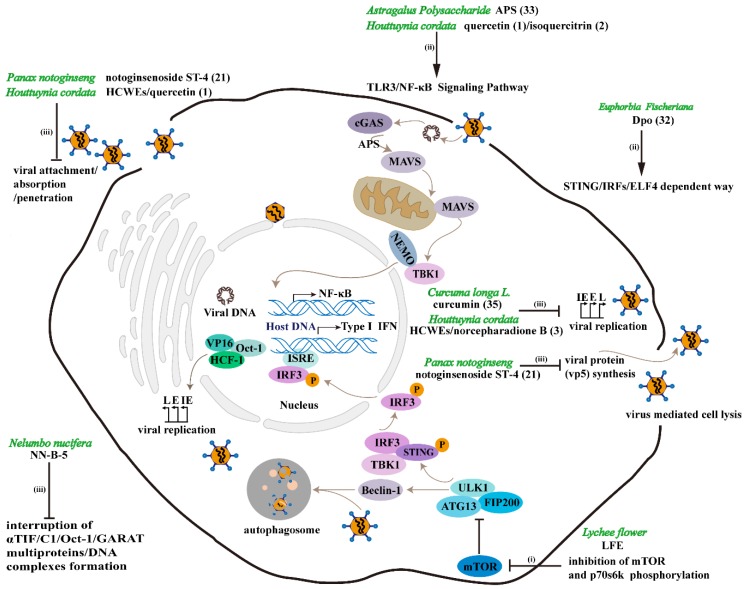
Pharmacodynamic mechanism of TCM against HSV-1. Anti-HSV-1 effects of TCM are mainly concentrated on the following three aspects: (i) TCM counteracts HSV-1 by regulating autophagy. mTOR (mammalian target of rapamycin) functions as a negative autophagy regulator. LFE can induce autophagy via decreasing the phosphorylation of mTOR and p70S6K (ribosomal p70S6 kinase). Consequently, Beclin-1 and LC3 (microtubule-associated protein 1 light chain 3)-II are activated, leading to autophagy-mediated clearance of HSV-1 [32]; (ii) TCM exerts anti-viral effects by enhancing immunity. For instance, as ingredients in the HCWEs, quercetin (**1**) and isoquercitrin (**2**) inhibit *NF-κB* (nuclear factor-kappa B) activation [42]. Dpo (**32**) also improves the organism’s immunity against HSV-1 in a STING-dependent manner, hence contributing to a sustained and significant increase in *IRF7* (interferon regulatory factor 7) [59]. Similarly, APS (**33**) (astragalus polysaccharide) promots immunological function by markedly increasing the expression of *TNF-α* (tumor necrosis factor-α), *IL-6* (interleukin-6), *TLR3* (toll-like receptor 3), and *NF-κB* provoked by HSV-1 [60]. From another perspective, IRF3 (interferon regulatory factor 3) can also be phosphorylated, and phosphorylated IRF3 activated type I interferon expression by binding to ISRE (interferon-stimulated response element) [76]. However, after autophagy-dependent STING delivery of TBK1 (TANK-binding kinase 1) to endosomal/lysosomal compartments, provoked ULK1 (unc-51 like autophagy activating kinase 1) can subsequently inhibit STING (Stimulator of Interferon Genes) function by phosphorylation S366, and IRF3 function is suppressed, thus preventing the persistent transcription of innate immune genes [77]. Mitochondria, the main organelles in eukaryotic cells, play an important role in antiviral process partially due to the mitochondrial localiazation of MAVS (Mitochondrial antiviral-signaling protein). Interestingly, the NEMO (the regulatory subunit of the IKK complex) dependent cGAS-MAVS-TBK1 signaling pathway is essential for *IRF3* and *NF-κB* activation [78]; and (iii) TCM exerts antiviral effects by inhibiting HSV-1 replication or inactivation of HSV-1 in the process of viral attachment/absorption/penetration. HCWEs and quercetin (**1**) inhibit the infection of HSV-1 via blocking of viral binding and penetration. Additionally, HCWEs, Norcepharadione B (**3**), and curcumin (**35**) can inhibit viral replication [42,45,75]. Notoginsenoside ST-4 (**21**) prevents HSV-1 from penetrating into cells and effectively blocks the synthesis of vp5 [53]. NN-B-5 interrupts the formation of αTIF/C1/Oct-1/GARAT multiprotein/DNA complexes, resulting in reduced expression of ICP0 (infected cell polypeptide 0) and ICP4 [35]. HSV-1, which can escape from various antiviral pathways, can cascade linearly and express immediate early (IE), early (E), and late (L) genes. After expression of the L gene, the cells produce a large number of mature viral particles, causing the cells to rupture and die. Bold numbers in parentheses refer to the numbers of corresponding compounds.

In recent years, biotechnology has been widely applied to all areas of research and production of TCM. The pharmacodynamic mechanism of TCM has been gradually revealed, as a result of the development of molecular biological methods. The anti-HSV-1 effects of TCM are mainly concentrated on the following three aspects: (i) TCM counteracts HSV-1 by regulating autophagy; (ii) TCM exerts anti-viral effects by enhancing immunity; and (iii) TCM exerts antiviral effects by inhibiting HSV-1 replication or inactivation of HSV-1 in the process of viral attachment/absorption/penetration (see Figure 2, Table 1 and Table 3 for details).

### 4.1. TCM Resists HSV-1 by Enhancing Organism Immunity

It is well known that innate immunity plays an important role in the antiviral process. Compared with chemical drugs, TCM exerts its effect by enhancing immunity. Clinical studies have demonstrated that TCM greatly improved the immunity of HIV positive AIDS (acquired immunodeficiency syndrome) patients and alleviated the side effects. Whether the anti-HSV-1 mechanism of TCM relates to the enhancement of immunity is a question to be answered.

Accordingly, Dpo (**32**), isolated from *Euphorbia fischeriana* Steud, up-regulated immunity to counteract HSV-1. The level of IRF7 rose consistently and dramatically during HSV-1 infection after the treatment of Dpo (**32**). However, Dpo (**32**) failed to restrict HSV-1 replication in *IRF3* or *IRF7* deficient macrophages. Since *IRF7* was an interferon stimulated gene (*ISG*), the *ISG* regulation might be important and should be considered carefully. Interestingly, experimental study of *STING* knockout mice uncovered that Dpo (**32**) could only exert its antiviral activity in the presence of *STING*. This further suggested that Dpo (**32**) activated the immunity to resist the virus by up-regulating *ISGs* and inflammatory genes in a type I IFNs independent manner [59]. AqMOL remarkably limited the development of herpetic skin lesions and reduced virus titers in the brain on day 4, with no toxicity shown. Further studies on the mechanism have found that augmentation of the delayed-type hypersensitivity response by AqMOL might contribute to its pesticide effect on HSV-1 infection [44]. Similarly, astragalus polysaccharide (APS, **33**), the most immunoreactive substance extracted from Astragalus, are commonly used in immune related diseases. Lately, the antiviral effect of APS was detected in astrocytes infected by HSV-1. Research uncovered that APS could not inhibit the virus directly, but instead it protected astrocytes by promoting immunological function, such as markedly increasing the expression of tumor necrosis factor-α (TNF-α), interleukin6 (IL-6), Toll-like receptor (TLR3), and nuclear factor-κB (NF-κB) provoked by HSV-1 [60].

### 4.2. TCM Exerts Anti-HSV-1 Effect by Inducing Autophagy

In addition to immunity enhancement, the regulation of autophagy also links to the anti-HSV-1 effect and this has become a field of interest for scientists. As a conserved physiological process, autophagy not only plays an important role in maintaining cellular homeostasis, but also participates in many crucial physiological processes, such as the elimination of exogenous microorganisms, antigen presentation, and inherent immune response.

As mentioned above, LFE decreased the cell viability of HSV-1 infected SIRC cells. Considering the role of the mammalian target of rapamycin (mTOR) in the regulation of cell proliferation and protein synthesis, the expression of mTOR and its downstream target, ribosomal p70S6 kinase (p70S6K), were evaluated in HSV-1 infected SIRC cells and in the LFE pretreated groups. Results revealed that LFE partially decreased the phosphorylation of mTOR and p70S6K32 [32]. It has been reported that autophagy may resist HSV-1 infection by presenting its antigen on major histocompatibility complex I (MHC-I) [79]. Consequently, the expression of Beclin -1 and LC3-II were also analyzed and the expression level was shown to be higher in the LFE pretreated groups than in the HSV-1 infected group. In sum, LFE inhibited the replication of HSV-1 by down-regulating the phosphorylation of mTOR and p70S6K, leading to the induction of Beclin-1 and LC3-II. Most of the current anti-HSV-1 therapies are initiated only after the appearance of symptoms, and HSV-1 will become drug-resistant after long-term application of a certain kind of chemical drugs, hence pretreating a patient, with a high risk of HSV-1 reactivation due to a compromised immune system and high level of stress, with LFE may be clinically useful [32].

However, certain viruses can enhance autophagy or exploit autophagy for replication and pathogenesis [80]. It could be seen that autophagy played a double-edged role in the process of HSV-1 infection. Further research of TCM with anti-HSV-1 activities based on autophagy machinery will still be needed.

### 4.3. TCM Exerts Antiviral Effects by Inhibiting HSV-1 Replication or Inactivation of HSV-1

Other natural products inhibit HSV-1 by impairing the replication of viral genes. Fraction A in the crude ethanol extract of *Antrodia camphorate* significantly suppressed HSV-1 replication in Vero cells after being infected with HSV-1 for 1 to 4 h [34]. Nevertheless, further studies are required to find out the molecular mechanism and signal pathway mining of compound A against HSV-1. Time course results indicated that the inhibitory effect of NN-B-5 on HSV-1 was the result of viral replication inhibition, instead of cytotoxicity, arrested cell growth, or viral adsorption blocking. The mechanism of the antiviral effect of NN-B-5 was then studied in vitro. The result of electrophoretic mobility shift assay (EMSA) indicated that NN-B-5 reduced ICP0, and ICP4 might be related to an interruption in the formation of αTIF/C1/Oct-1/GARAT multiprotein/DNA complexes [35]. RT-PCR analysis showed that *UL30* and *UL39* (two important delayed early genes of HSV-1 genome) and *US6* (a late HSV-1 gene) were all suppressed by alkaloids extracted from *Tripterygium hypoglaucum*, individually with the inhibiting efficacy at 74.6%, 70.9%, and 62.6%, respectively, at the concentration of 12.5 μg/mL [36]. Specifically, in the presence of notoginsenoside ST-4 (**21**), the penetration of HSV-1 into the cell membrane was prohibited and no viral capsid protein (referred as vp5) synthesis was observed in HSV-1 infected cells following the treatment of 10 μM notoginsenoside ST-4 (**21**) at 2 h post-infection [53].

Many theories exist to explain the antiviral mechanism of emodin (**22**). Previous studies have shown that emodin (**22**) might play an antiviral role by inhibiting casein kinase2 (CK2), which in turn phosphorylate a lot of viral proteins, which are necessary for the life cycle of the virus [69,70]. Alves et al. demonstrated that emodin (**22**) might disrupt the lipid bilayer to inactivate the virus [68]. Another study illustrated that emodin (**22**) could specifically inhibit UL12, an HSV-1 protein involved in DNA processing and capsid egression [67]. Recently, researchers evaluated the efficacy of emodin (**22**) using ACV as a positive control. It was found that pretreating HEp-2 cells with emodin (**22**) before HSV-1 infection was unable to block viral adsorption [54]. Zhao and coworkers carried out other studies on isolated compounds and reported that protocatechuyl aldehyde (**30**) resisted HSV-1 by inhibiting viral replication as well [58]. The similar trend of inhibition on viral replication indicated that caffeic acid (**27**) exerted its antiviral efficacy by inhibiting the replication of HSV-1 rather than adsorption [57].

All evidence implies that the pharmacodynamic mechanisms of OB and ACV are likely to be different. Additional time assay was conducted to compare the anti-HSV-1 activity of 1246TGG and ACV at different stages during the viral growth cycle. The results revealed that 1246TGG suppressed viral growth mainly within 3 h post-infection, as opposed to 3–6 h post-infection for ACV. Viral DNA was isolated and the copy number of HSV-1 was examined. Data indicated that the replication of HSV-1 DNA was dramatically reduced with the prior treatment of 1246TGG. Immunofluorescence staining also showed that 1246TGG could suppress the expression of gB (glycoprotein B). To sum up, 1246TGG not only directly inactivates the virus at an early stage of infection, leading to the blockage of viral attachment and penetration, but also inhibits viral biosynthesis by suppressing gene expression (*UL52* and *UL27*), viral DNA replication, and gB synthesis. Nevertheless, RNA synthesis of IE (*UL54*) gene was not inhibited by1246TGG [55].

Direct inactivation of HSV-1 is one of the antiviral mechanisms of TCM. Time-of-addition effect of YCHT water extract on HSV-2 revealed that there are two methods to protect cells from infection: Concurrent addition of virus with YCHT water extract and addition of YCHT water extract at 2 h post-infection. YCHT is required to remain present for the entire process for both methods. Additionally, the pre-treatment of HSV-2 with YCHT water extract could also irreversibly reduce the infection of HSV-2, which suggested that the mechanism of action of YCHT might be virus inactivation [39].

### 4.4. Natural Anti-HSV-1 Products with Unclear Mechanism

Although the mechanisms of many TCM against HSV-1 have been elucidated, there are other extracts or compounds with unknown mechanisms. A report by Pengcuo et al. uncovered that alantolactone (**34**) at a concentration of 10^−7^ g/mL inhibited viral infection remarkably [61]. Nevertheless, the antiviral effect of alantolactone (**34**) in vivo and the underlying mechanism deserves further study. Zhang et al. reported that compounds extracted from the roots of *Ilex asprella* exhibited interesting anti-HSV-1 activity. At present, asprellanoside A (**38**) and oblonganoside H (**39**) show anti-HSV-1 activity [63]. Polyphenolic compounds extracted from *Agrimonia pilosa*, *Pithecellobium clypearia*, and *Punica granatum* also showed anti-HSV-1 activity [81]. In another report, ursolic acid exhibited activity against HSV-1. Although the SI values for ursolic acid and apigenin were lower than that of ACV, both compounds can be used as potential clinical drugs in the treatment of ACV-resistant patients [37]. Acacetin-7-*O*-[6′′′-*O*-acetyl-β-d-galactopyranosyl-(1→2)]-β-d-glucopyranoside) (**24**) and 2,5-dihydroxybenzoic acid (**25**) extracted from *O. vulgare* showed weak anti-HSV-1 activity [56]. Water extract of *P.* major exhibited a slight anti-HSV-1 activity, however, the pure compound, namely caffeic acid (**27**), showed the strongest activity against HSV-1. Similarly, three bisbenzylisoquinoline alkaloids, like aromoline, (−)-norcycleanine, and obamegine, exhibited antiviral activities in Vero cells. Nevertheless, none of them showed any activity against HSV-1 when mice were orally administered low dosages. When FK-3000 (**31**) was administered at a dose of 10, 25 mg/kg, p.o. for 10 consecutive days, symptoms of HSV-1 infection were significantly alleviated and survival time was extended [40].

Although the mechanism of these compounds against HSV-1 has not been clarified, preliminary experimental data have shown that these compounds have the potential to be developed as anti-HSV-1 drugs.

## 5. Advantages and Limitations of TCM in the Prevention and Treatment of HSV-1 Infection

Although the research on TCM against HSV-1 is mainly conducted in vitro or in mouse models, hence no clinical data was generated, TCM serve as a treasure house for exploring natural products with anti-HSV-1 activity. Also, natural products can regularly be used as structural sources for new compounds, so as to provide strategies for the development of new anti-HSV-1 drugs.

To date, drugs used in the treatment of HSV-1 are mainly targeted at viral DNA polymerase and synthetic nucleotides. These current drugs cannot overcome the issues of drug resistance and re-activation from latency of HSV-1. “Preventing illness before it begins” is the classical view of TCM. This scientific thought of “prevention before illness, prevention of disease and change” has been summed up by ancient physicians in the course of preventing and treating plague for thousands of years, and is the model of health medicine dedicated to mankind by TCM. TCM has been widely used in the treatment of human infectious diseases. As one of the main sources of natural active products, in recent years, extracts or compounds with anti-HSV-1 activity have been gradually excavated from TCM. The premise of “preventing a disease before it becomes an illness” applies to anti-HSV-1 drugs as they can effectively improve immunity, making HSV-1 insensitive or latent HSV-1 difficult to be reactivated. This concept has been well documented in the existing anti-HSV-1 TCM that influence the innate immune pathway.

Consequently, the application of TCM to patients infected with HSV-1 to prevent the recurrence of latent HSV-1 should be encouraged.

## 6. Concluding Remarks

The study of these TCM and natural products mainly focused on direct antiviral activity at different stages of virus infection (including viral attachment/absorption/penetration, viral replication, virus mediated cell lysis, etc.). However, these mechanisms were not enough to reflect the holistic view of TCM. There are many factors that lead to the susceptibility of HSV-1. Emotion has become an extremely important factor to determine the occurrence, development, and rehabilitation of the disease due to rapid living pace, ever-increasing work stress, and deterioration of the living environment. The TCM theory has recognized emotional pathogeny and its increasingly more important value and significance in the etiology of modern diseases. Our preliminary research has shown that emotional stress increased the susceptibility to influenza virus [82,83,84]. Hence, TCM might function as a potential source for HSV-1 therapy by indirect antiviral activities (reducing the susceptibility to HSV-1). In addition, oxidative stress seems closely related to HSV-1 infection. It has been revealed that stimulation with HSV-1 elevated intracellular ROS (reactive oxygen species) in microglial cells via activation of p38 MAPK and p42/p44 ERK [85]. Downregulating cellular NF-κB and MAPK pathways induced by oxidative stress could block HSV infection [86]. However, whether oxidative stress is the cause of HSV-1 susceptibility or the result of HSV-1 infection will be one of the future directions of HSV-1 research. Moreover, the most distinct feature of HSV-1 is that once it is infected, it will be latent in the host for life. Subsequently, in addition to reducing the susceptibility of the human body to HSV-1, how to prevent the re-activation of HSV-1 in host is also an important issue in current research. More importantly, stress is related to the re-activation of latent HSV-1, but the specific molecular mechanism remains unclear.

The relationship between autophagy and HSV-1 is in a dynamic equilibrium state. On the one hand, autophagy was beneficial for the removal of HSV-1. It has been revealed that ICP34.5 (infected cell polypeptide 34.5) inhibited *Beclin-1* mediated autophagy by targeting Beclin-1 and alleviated the clearance of HSV-1 by host cells through autophagy [87]. Additionally, TBK1 promoted autophagy by phosphorylating autophagy receptor protein P62 (sequestosome 1) and OPTN (optineurin). ICP34.5 inhibited the removal of HSV-1 by *TBK1*-induced autophagy through interactions with TBK1 [88,89]. On the other hand, another theory stated that autophagy could promote the replication of HSV-1. It has been reported that *MyD88* (myeloid differentiation primary response 88) mediated autophagy was very important for the replication of HSV-1 in THP-1 cells during the process of HSV-1 infection. The replication of HSV-1 was inhibited in THP-1 cells with the treatment of either autophagy inhibitor spautin-1 and 3-MA (3-Methyladenine), or *Beclin-1* interference by siRNA. This suggested that autophagy was beneficial for the replication of HSV-1 [90]. A comprehensive understanding of the roles of autophagy during viral infection will contribute to the development of anti-HSV-1 drug targets.

The emergence of drug resistant viral strains encourages the search for novel antiviral drugs, including new chemical drugs, TCM, marine drug, etc. Drug sources and drug resistance have always been the restraining factors in the research and development of anti-HSV-1 drugs. Several compounds have been already identified to inhibit HSV-1 in vitro and/or in vivo, thus efficient synthesis should be developed to produce enough bioactive agents to meet the needs of clinical researches. Opening new areas of resources, and exploring new methods and techniques are effective ways in solving the problem of drug source.

Since no suitable drug is available for the treatment of latent HSV-1 infection in clinics yet, searching for strategies based on the prevention of transmission, suppression of reactivation, and viral shedding together with inhibition of epithelial damage is an effective measure to get out of the predicament of drug research and development against HSV-1. Moreover, some mutated HSV-1 strains produced by the CRISPR/Cas9 system could be potential vaccines in full swing for the prevention of HSV-1 in humans [91,92].

Finally, modern experimental methods help to identify molecular targets of natural products, such as gene expression microarray and high-throughput screening techniques. They also help to screen for new natural small molecular compounds against HSV-1. In addition, sequencing the HSV-1 genome will help to identify the basic genes for virus survival and corresponding encoded protein, which can be used as a molecular target for the development of new anti-HSV-1 drugs. Digging for mutated viral genes between normal virus and drug-resistant virus by sequencing will also provide a new direction for the development of anti-HSV-1 drugs.

## Figures and Tables

**Table 1:** Plant extracts with potential anti-HSV-1 (Herpes simplex virus type 1) activities.

Source	Extracts	Target/Mechanism	IC_50_ (μg/mL)	CC_50_ (μg/mL)	In Vitro	In Vivo	HSV-1 Strain	MOI	References
Lychee flower	Water and ethanol	Inhibition of mTOR and p70s6k phosphorylation	Not mentioned	Not mentioned	√	×	Not mentioned	1 pfu/cell	[32]
*Moringa oleifera*	Ethanol	Not mentioned	100.0 ± 5.3	875 ± 35	√	√	7401H	100 pfu/0.2 mL (60 mm dishes)	[33]
*Ventilago denticulata*	Ethanol	Not mentioned	46.3 ± 1.5	838 ± 53	√	√	7401H	100 pfu/0.2 mL (60 mm dishes)	[33]
*Antrodia camphorata* mycelia	Crude extract Fraction A Fraction B	Not mentioned	61.2 ± 5.5 8.2 ± 1.80 120.0 ± 3.5	485.0	√	×	F	2 pfu/cell	[34]
				197.0					
				235.0					
*Nelumbo nucifera*	NN-B-5	Interruption of αTIF/C1/Oct-1/GARAT multiproteins/DNA complexes formation	21.3 ± 1.6	Not mentioned	√	×	KOS/TK-HSV-1	100 pfu/well	[35]
*Tripterygium hypoglaucum*	Total alkaloids	Not mentioned	6.5	46.6	√	×	SM44	100 TCID_50_	[36]
*Ocimum basilicum*	Water Ethanol	Not mentioned	90.9 ± 2.6 108.3 ± 2.4	1469.3 684.8	√	×	KOS	20 TCID_50_	[37]
Almond skin	Methanol	Inhibition of viral adsorption and blocking the production of viral particles	Not mentioned	Not mentioned	√	×	F/VP26GFP-HSV-1	1 pfu/cell	[38]
Yin Chen Hao Tang (YCHT)	Water	Not mentioned	142.5 ± 1.7	850.7 ± 1.7	√	×	KOS	100 pfu/well	[39]
*Stephania cepharantha*	Methanol	Not mentioned	18	Not mentioned	√	√	7401H	100 pfu/0.2 mL (60 mm dishes)	[40]
	CHCl_3_-soluble fraction (alkaloid raction)		8						
*Houttuynia cordata*	Water	Not mentioned	822.39	>1000	√	×	Not mentioned	Not mentioned	[41]
*Houttuynia cordata*	Water	Inhibition of NF-κB activation and blocking viral binding/penetration/replication	692	>100,000	√	×	F	1 pfu/cell	[42]

CC_50_, concentration that reduces the growth of target cells by 50%; IC_50_, inhibitory concentration of compound that produces 50% inhibition of virus-induced cytopathic effects; MOI, the infection of HSV-1 at a multiplicity of infection. √, relevant information could be queried in the article; ×, no relevant information was descripted.

**Table 2:** The anti-HSV-1 activities of pure compounds from *H. cordata.*

Compounds	Type	Target/Mechanism	IC_50_ (μg/mL)	CC_50_ (μg/mL)	In Vitro	In Vivo	HSV-1 Strain	MOI	References
Quercetin (**1**)	Flavonoid	Inhibition of NF-κB activation and viral entry	52.9	>100,000	√	×	F	1 pfu/cell	[42]
Isoquercitrin (**2**)		Inhibition of NF-κB activation	0.42						
Norcepharadione B (**3**)	Alkaloid	Not mentioned	170 μM	Not mentioned	√	×	KOS	3 pfu/cell	[45]
Houttuynoid A (**4**)	Flavonoid	Not mentioned	23.50 ± 1.82	166.38	√	×	Not mentioned	Not mentioned	[46]
Houttuynoid B (**5**)			57.71 ± 8.03	181.79					
Houttuynoid C (**6**)			50.75 ± 11.07	531.35					
Houttuynoid D (**7**)			59.89 ± 6.63	180.87					
Houttuynoid E (**8**)			42.03 ±10.22	134.92					
Houttuynoid F (**9**)	Flavonoid	Not mentioned	Not mentioned	Not mentioned	√	×	Blue	Not mentioned	[47]
Houttuynoid G (**10**)	Flavonoid	Not mentioned	38.46 ± 9.57	113.10 ± 12.16	√	×	Blue	0.5 pfu/cell	[48]
Houttuynoid H (**11**)			14.10 ± 0.11	44.55 ± 4.63					
Houttuynoid I (**12**)			62.00 ± 2.06	63.06 ± 8.34					
Houttuynoid J (**13**)			70.76 ± 2.22	100.87 ± 6.14					
Houttuynoid K (**14**)	Flavonoid	Not mentioned	Not mentioned	Not mentioned	√	×	Blue	Not mentioned	[47]
Houttuynoid L (**15**)									
Houttuynoid M (**16**)	Flavonoid	Not mentioned	17.72	>200	√	√	Blue/F	0.5 pfu/cell	[49]
Houttuynoid A (**4**)			12.42					Not mentioned	
Houttuynoid A (**4**)	Flavonoid	Not mentioned	23.50 ± 1.82	166.36 ± 9.27	√	√	Blue/F	0.5 pfu/cell	[50]

CC_50_, concentration that reduces the growth of target cells by 50%; IC_50_, inhibitory concentration of compound that produces 50% inhibition of virus-induced cytopathic effects; MOI, the infection of HSV-1 at a multiplicity of infection. √, relevant information could be queried in the article; ×, no relevant information was descripted. Bold numbers in parentheses refer to the numbers of corresponding compounds.

**Table 3:** The anti-HSV-1 activities of pure compounds from TCM (traditional Chinese medicine).

Source	Compounds	Type	Target/Mechanism	IC_50_ (μg/mL)	CC_50_ (μg/mL)	In Vitro	In Vivo	HSV-1 Strain	MOI	References
*Radix isatidis*	3-(furan-2-yl)-7-hydroxyisoquinolin-1(2*H*)-one (**17**)	Aglycone derivative	Not mentioned	15.3	90.9	√	×	Not mentioned	100 TCID_50_/mL, 20 μL/well	[51]
	3-(Furan-2-yl)-7-(((2S,3R,5S,6R)-3,4,5-trihydroxy-6-(hydro-xymethyl)tetrahydro-2*H*-pyran-2-yl)oxy) isoquinolin-1(2*H*)-one (**18**)	Glucoside derivative	Not mentioned	42.4	72.1	√	×	Not mentioned	100 TCID_50_/mL, 20 μL/well	
	3-(5-(Hydroxymethyl)furan-2-yl)-7-(((2S,3*R*,5*S*,6*R*)-3,4,5-trihydroxy-6-(hydroxymethyl)tetrahydro-2*H*-pyran-2-yl)oxy)isoquinolin-1(2*H*)-one (**19**)	Isoquinoline derivative	Not mentioned	79.1	619.4	√	×	Not mentioned	100 TCID_50_/mL, 20 μL/well	
*Strobilanthes cusia*	Lupeol (**20**)	Triterpenoid	Not mentioned	11.70	49.3	√	×	KOS	100 pfu/cell	[52]
*Panax notoginseng*	notoginsenoside ST-4 (**21**)	Dammarane-type saponin	HSV-1 penetration and viral protein (vp5) synthesis	16.47 ± 0.67	510.64 ± 4.56	√	×	F	30 pfu/well	[53]
*Rheum tanguticum*	emodin (**22**)	Anthraquinone derivative	Not mentioned	Not mentioned	Not mentioned	√	√	F	100 TCID_50_/mL	[54]
*Phyllanthus emblica*	1,2,4,6-tetra-*O*-galloyl-β-d-glucose (1246TGG) (**23**)	Polyphenolic	Not mentioned	10.77 ± 0.61	>253.63	√	×	Not mentioned	30 pfu/well (24-well plates)	[55]
*Origanum vulgare*	acacetin-7-*O*-[6′′′-*O*-acetyl-β-d-galactopy-ranosyl-(1→2)]-β-d-glucopyranoside (**24**)	Phenolic compound	Not mentioned	38.5	Not mentioned	√	×	F	100 TCID_50_, 100 μL	[56]
	2,5-dihydroxybenzoic acid (**25**)			32.7						
*Plantago major*	chlorogenic acid (**26**)	Phenolic compound	Not mentioned	47.6	3995	√	×	KOS	0.002–0.025 pfu/cell	[57]
	caffeic acid (**27**)	Phenolic compound		15.3	10,293					
	baicalein (**28**)	Flavonoid		4.7	19.5					
	vanillic acid (**29**)	Phenolic compound		88.1	1338					
*Ranunculus sceleratus*	protocatechuyl aldehyde (**30**)	Phenolic aldehyde	Not mentioned	17.34 ± 1.2	>200	√	×	Not mentioned	100 pfu/well	[58]
*Stephania cepharantha*	FK-3000 (**31**)	Alkaloid	Not mentioned	7.8	Not mentioned	√	√	7401H	100 pfu, 60 mm dishes	[40]
*Euphorbia Fischeriana*	Dpo (**32**)	Not mentioned	STING/IRFs/ELF4 dependent way	Not mentioned	Not mentioned	×	√	Not mentioned	Not mentioned	[59]
*Astragalus*	astragalus polysaccharide (**33**)	Polysaccharide	TLR3/NF-κB Signaling Pathway	Not mentioned	120	√	×	SM44	Not mentioned	[60]
*Inulae Radix* (Tu-Mu-Xiang)	alantolactone (**34**)	Sesquiterpene lactone	Not mentioned	0.04	>1	√	×	Not mentioned	Not mentioned	[61]
*Curcuma longa* L.	curcumin (**35**)	Phenolic	Not mentioned	33.0	484.2	√	×	KOS	100 TCID_50_	[62]
	gallium-curcumin (**36**)			13.9	255.8					
	Cu-curcumin (**37**)			23.1	326.6					
*Ilex asprella*	asprellanoside A (**38**)	Triterpenoid Saponin	Not mentioned	140	Not mentioned	√	×	F	40 pfu/well	[63]
	oblonganoside H (**39**)			180						

CC_50_, concentration that reduces the growth of target cells by 50%; IC_50_, inhibitory concentration of compound that produces 50% inhibition of virus-induced cytopathic effects; MOI, the infection of HSV-1 at a multiplicity of infection. √, relevant information could be queried in the article; ×, no relevant information was descripted. Bold numbers in parentheses refer to the numbers of corresponding compounds.

## Abbreviations

HSV-1	Herpes simplex virus type 1
ACV	Acyclovir
TCM	Traditional Chinese Medicine
LFE	Lychee flower extract
mTOR	Mammalian target of rapamycin
p70S6K	p70S6 kinase
MHC-I	Major histocompatibility complex I
AqMOL	Aqueous extract
EMSA	Electrophoretic mobility shift assay
ISG	Interferon stimulated genes
CPE	Cytopathic effect
APS	Astragalus polysaccharide
TNF-α	Tumor necrosis factor-α
IL-6	Interleukin 6
TLR3	Toll-like receptor 3
NF-κB	Nuclear factor-κb
1246TGG	1,2,4,6-tetra-*O*-galloyl-β-d-glucose
OB	Ocimum basilicum
SI	Selectivity index
YCHT	Yin Chen Hao Tang
HCWEs	*H. cordata* water extracts
